# Supplementary material for: Detailed molecular and epigenetic characterization of the pig IPEC-J2 and chicken SL-29 cell lines
Source: iScience. 2023 Feb 20;26(3):106252. doi: 10.1016/j.isci.2023.106252 (PMC10018572; doi:10.1016/j.isci.2023.106252)
Supplement: Data S2. Complete homer output for identified motifs in Chicken SL-29, related to Tables 5 and 6 — Homer motif analysis results for histone modifications H3K4me1, H3K4me3, H3K27ac, enhancers, and ATAC-seq of chicken SL-29 cell line. Parameters for possible false positives is as mentioned earlier for S5. [file mmc3.zip › Data_S2/S6/Chicken_SL_29/peak_files_H3K27ac/homerResults/motif2.similar.html]

motif2

## Information for motif2

T
C
A
G
T
A
G
C
T
A
G
C
C
G
T
A
A
C
G
T
T
A
C
G
C
A
T
G
A
G
T
C
A
T
C
G
C
T
A
G
A
T
G
C
A
T
C
G
T
A
C
G
  
Reverse Opposite:  

A
T
G
C
T
A
G
C
T
A
C
G
G
A
T
C
A
T
G
C
A
C
T
G
G
T
A
C
A
T
G
C
C
G
T
A
C
G
A
T
A
T
C
G
A
T
C
G
A
G
T
C
  

|  |  |
| --- | --- |
| p-value: | 1e-78 |
| log p-value: | -1.818e+02 |
| Information Content per bp: | 1.618 |
| Number of Target Sequences with motif | 1823.0 |
| Percentage of Target Sequences with motif | 6.05% |
| Number of Background Sequences with motif | 1147.3 |
| Percentage of Background Sequences with motif | 3.80% |
| Average Position of motif in Targets | 149.8 +/- 93.2bp |
| Average Position of motif in Background | 150.8 +/- 93.9bp |
| Strand Bias (log2 ratio + to - strand density) | 0.1 |
| Multiplicity (# of sites on avg that occur together) | 1.11 |
| Motif File: | file (matrix) reverse opposite |

### Similar de novo motifs found

|  |  |  |  |  |  |  |  |
| --- | --- | --- | --- | --- | --- | --- | --- |
| Rank | Match Score | Redundant Motif | P-value | log P-value | % of Targets | % of Background | Motif file |
| 1 | 0.858 | T G A C T A G C C T G A A C G T A C T G A C T G A G T C A T C G | 1e-75 | -173.382700 | 9.17% | 6.42% | motif file (matrix) |
| 2 | 0.878 | G A C T T A G C C A T G T A G C G A T C A C T G A G T C A G T C G C T A A G C T | 1e-67 | -155.917043 | 9.76% | 7.05% | motif file (matrix) |
| 3 | 0.713 | A C G T A C T G A C T G A G T C A C T G A C T G C G T A A C T G A C T G A G T C | 1e-16 | -38.659900 | 0.18% | 0.04% | motif file (matrix) |
| 4 | 0.607 | A C G T A G T C A G T C A C T G A G T C A C T G C G T A C G T A | 1e-7 | -17.368947 | 0.21% | 0.10% | motif file (matrix) |
